# End of 2022/23 Season Influenza Vaccine Effectiveness in Primary Care in Great Britain

**DOI:** 10.1111/irv.13295

**Published:** 2024-05-14

**Authors:** Heather J. Whitaker, Naoma Willam, Simon Cottrell, Rosalind Goudie, Nick Andrews, Josie Evans, Catherine Moore, Utkarsh Agrawal, Katie Hassell, Rory Gunson, Jana Zitha, Sneha Anand, Praveen Sebastian‐Pillai, Panoraia Kalapotharakou, Cecilia Okusi, Katja Hoschler, Gavin Jamie, Beatrix Kele, Mark Hamilton, Anastasia Couzens, Catherine Quinot, Kathleen Pheasant, Rachel Byford, Kimberly Marsh, Chris Robertson, Simon de Lusignan, Christopher Williams, Maria Zambon, Jim McMenamin, Conall H. Watson

**Affiliations:** ^1^ Statistics, Modelling and Economics Department UK Health Security Agency London UK; ^2^ Clinical and Protecting Health Public Health Scotland Glasgow UK; ^3^ Public Health Wales Communicable Disease Surveillance Centre Public Health Wales Cardiff UK; ^4^ Nuffield Department of Primary Care Health Sciences University of Oxford Oxford UK; ^5^ Immunisation and Vaccine Preventable Diseases Division UK Health Security Agency London UK; ^6^ Wales Specialist Virology Centre Public Health Wales Microbiology Cardiff UK; ^7^ West of Scotland Specialist Virology Centre NHS Greater Glasgow and Clyde Glasgow UK; ^8^ Respiratory Virus Unit UK Health Security Agency London UK; ^9^ Department of Mathematics and Statistics University of Strathclyde Glasgow UK; ^10^ Research and Surveillance Centre Royal College of General Practitioners London UK

**Keywords:** effectiveness, influenza, vaccine

## Abstract

**Background:**

The 2022/23 influenza season in the United Kingdom saw the return of influenza to prepandemic levels following two seasons with low influenza activity. The early season was dominated by A(H3N2), with cocirculation of A(H1N1), reaching a peak late December 2022, while influenza B circulated at low levels during the latter part of the season. From September to March 2022/23, influenza vaccines were offered, free of charge, to all aged 2–13 (and 14–15 in Scotland and Wales), adults up to 49 years of age with clinical risk conditions and adults aged 50 and above across the mainland United Kingdom.

**Methods:**

End‐of‐season adjusted vaccine effectiveness (VE) estimates against sentinel primary‐care attendance for influenza‐like illness, where influenza infection was laboratory confirmed, were calculated using the test negative design, adjusting for potential confounders.

**Methods:**

Results In the mainland United Kingdom, end‐of‐season VE against all laboratory‐confirmed influenza for all those > 65 years of age, most of whom received adjuvanted quadrivalent vaccines, was 30% (95% CI: −6% to 54%). VE for those aged 18–64, who largely received cell‐based vaccines, was 47% (95% CI: 37%–56%). Overall VE for 2–17 year olds, predominantly receiving live attenuated vaccines, was 66% (95% CI: 53%–76%).

**Conclusion:**

The paper provides evidence of moderate influenza VE in 2022/23.

## 1 Introduction

During the 2020/21 and 2021/22 seasons, amid the COVID‐19 pandemic, relatively little influenza activity was detected in the United Kingdom [1, 2], while 2022/23 saw the return of influenza to prepandemic levels [3]. Indicators of influenza activity during November–December 2022, while vaccine rollout was still underway, suggested higher levels of early season circulation than that seen from 2017 to 2022. Influenza activity rose sharply to a peak during December 2022, which subsequently dropped off steeply during January 2023. This start to the influenza season was dominated by A(H3N2) with some A(H1N1)pdm09 cocirculation. From February 2023 to the end of the season, UK influenza activity was considerably lower, predominated by influenza B.

The United Kingdom now has a long running programme of influenza vaccination in those aged 65 years and older and those > 6 months of age at clinical risk for severe outcomes. Since the 2018/19 influenza season, adults aged 65 years and older have been preferentially offered adjuvanted inactivated egg‐grown influenza vaccines (aIIV), with recombinant vaccine (IIVr) an alternate first‐line vaccine [4]. Other groups offered seasonal influenza vaccines in 2022/23 include health and social care workers, care home residents, carers, close contacts of immunosuppressed individuals and pregnant women [5, 6, 7]. From 2020/21 to 2022/23, all adults aged 50–64 were additionally offered a seasonal influenza vaccine in an effort to minimise winter pressures on the health service during the COVID‐19 pandemic [8]; this was a temporary policy extension that was not carried through to the 2023/24 season. For the 2022/23 season, cell‐based vaccines (IIVc) or IIVr were recommended for adults aged 18–64 [6, 7, 8]; in practice, most vaccinators offered cell‐based vaccines. Rollout of influenza vaccines universally to children began in 2013/14 in the United Kingdom [9]. By 2019/20, all preschool and primary school children 2–10 years of age were offered live attenuated influenza vaccine (LAIV), or if unsuitable, an intramuscular vaccine [10]. This offer included secondary school age children aged 11–15 during 2022/23; in England, those aged 11–13 were prioritised depending on vaccine availability, whereas in Scotland and Wales, the offer was universal [6, 7, 8]. However, many older children in England were not vaccinated until early 2023, after the peak of influenza activity in December. All vaccines were quadrivalent in the 2022/23 season.

Influenza vaccine effectiveness (VE) varies from season to season depending on the specific strains in circulation and how well these match the vaccine strains that are selected months in advance. Although reported effectiveness of seasonal influenza vaccines against influenza A(H1N1)pdm09 and influenza B is often higher, reduced VE against A(H3N2) strains has been observed globally in several seasons [11]. Reasons for this may include the following: egg‐adaption of vaccine strains, mutation rate of A(H3N2), cocirculation of different virus subclades and host factors, such as early childhood imprinting and the impact of repeated vaccination [12].

The United Kingdom had a well‐established system to monitor influenza VE each season based upon sentinel swabbing in primary care [13]. The COVID‐19 pandemic interrupted this system as patients with respiratory symptoms requiring medical guidance were directed away from traditional primary care and advised to contact the national telephone triage service [14, 15]. Sentinel swabbing schemes have since been reinvigorated as patients returned to face‐to‐face primary care and through efforts to recruit more practices and introduce postal self‐swabbing. Influenza VE in mainland UK primary care can be estimated for the first time since 2019/20 and with greater precision. This paper presents the end‐of‐season 2022/23 VE findings for laboratory confirmed infection in primary care, focusing on three age groups for whom distinct vaccine types were recommended: children aged 2–17, adults aged 18–64 and adults aged 65+.

## 2 Methods

### 2.1 Study Design and Population

The test‐negative design (TND) was used to estimate VE, with the study undertaken in the registered population of three sentinel general practice surveillance networks across the United Kingdom, all of which undertake respiratory swabbing. The three schemes are the following: the Royal College of General Practitioners (RCGP) Research and Surveillance Centre (RSC) network (covering England) [16], Public Health Scotland (PHS) and Public Health Wales (PHW). The PHS scheme (the Community Acute Respiratory Infection [CARI] surveillance programme) aims to swab patients presenting in the community with an acute respiratory infection (ARI) with onset in the last 7 days, while the RCGP RSC and PHW schemes aim to swab patients presenting with ARI onset in the last 10 days. All swabs are tested for influenza using RT‐PCR, in addition to testing for SARS‐CoV‐2, respiratory syncytial virus (RSV), human metapneumovirus (hMPV) and other respiratory viruses, depending on the scheme.

The study period was 5 September 2022, to coincide with vaccine rollout, to 16 April 2023, by which point influenza positivity in community swabs had dropped to < 2%. The study population was composed of patients presenting to their general practitioner (GP) with ARI or influenza‐like illness (ILI), whom either the GP consented verbally and swabbed during the consultation, or the GP directed to a self‐swabbing postal service. Participating GPs invited persons to provide a swab for diagnosis, regardless of vaccination status, and to complete a standard questionnaire that included date of onset. The collection and analysis of swab forms according to positivity was undertaken as part of routine surveillance of clinical respiratory infections in the population.

### 2.2 Outcomes and Exposures

Cases were patients who presented with ARI symptoms and tested positive for seasonal influenza A or B virus by real‐time polymerase chain reaction (PCR) testing. Controls were patients with ARI symptoms who tested negative for influenza A or B virus and who also tested negative for SARS‐CoV‐2 [17].

Vaccine history, including date of vaccination, was obtained by PHW from GP records and patients during the consultation, by PHS from the national vaccination registry and by RCGP RSC from a combination of patient records and the national vaccination registry. Patients were defined as vaccinated if they were reported to have received the 2022/23 seasonal vaccine at least 14 days before swabs were collected. This was extended to 21 days for children to allow ample time for any LAIV virus detectability to cease.

### 2.3 Laboratory Methods

Respiratory swabs are collected from symptomatic patients and referred to the testing laboratory where molecular testing for influenza A and B was undertaken. Positive samples for influenza A were subtyped H3 or H1 and, where possible, lineages for influenza B typed to Victoria and Yamagata.

Influenza laboratory confirmation was undertaken using comparable, in‐house developed real‐time PCR methods for detection of circulating influenza A and B viruses across the three surveillance networks. Suitable influenza positive samples were further characterised by next‐generation sequencing of the haemagglutinin (HA) genes of influenza A(H1N1)pdm09, A(H3N2) and influenza B, based on PCR detection cycle threshold (Ct) values ≤ 32, respectively. All genetic characterisation data were generated by the UKHSA Respiratory Virus Reference Unit using full genome amplification protocols for influenza A and B for sequencing on an Illumina MiSeq or Illumina NextSeq instruments. Influenza virus genomes were assembled using an in‐house developed pipeline, genetic clade assignments were done using an in‐house developed script and confirmed using FluSurver. All influenza whole genomes and HA‐only or HA‐NA partial genomes were uploaded to GisAID [18].

### 2.4 Statistical Methods

Using a test negative design, with influenza laboratory results as the outcome and influenza vaccination status and adjustment variables as the linear predictors, the odds ratio (OR) of being vaccinated between cases and controls was used to calculate the VE as (1 − OR) × 100% [19]. Potential confounders adjusted for in the multivariable logistic regression model included week of swab (cubic spline), age group, surveillance scheme (England, Scotland and Wales) and clinical risk status. All analyses were stratified by age groups 2–17, 18–64 and ≥ 65 years. Additional analyses split by vaccine type, where known: LAIV within those aged 2–17 years; IIVc, IIVe or aIIV for those aged 18–64; and aIIV for those aged 65 years and above (insufficient data were available for IIVr).

**FIGURE 1:**
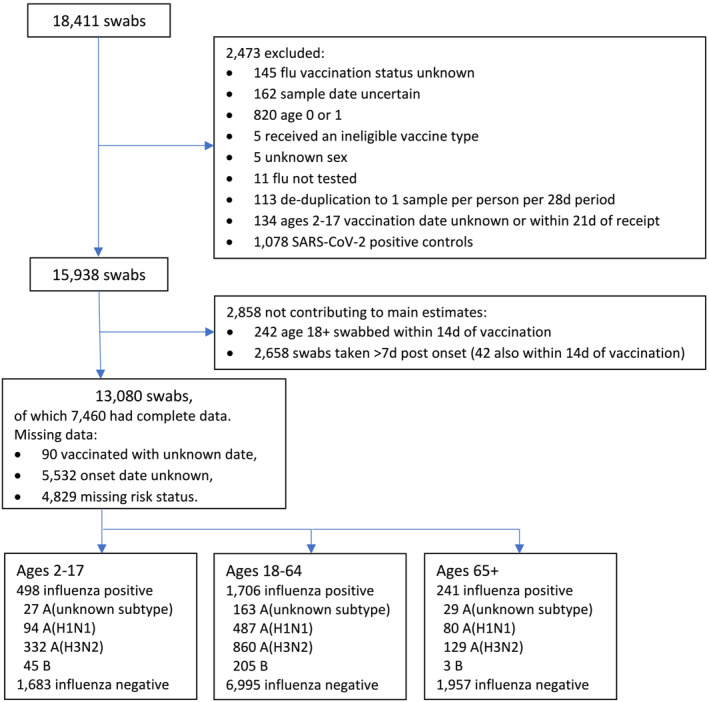
Flow diagram of swabs included in the study.

For inclusion in the study, we required a known flu vaccination status at the time of swabbing, age and sex and a definitive result for influenza. Patients were excluded if the timing of the swab was uncertain or where patients received a vaccine outside of national recommendations (e.g., LAIV in adults). SARS‐CoV‐2 positive controls were excluded due to the association between influenza vaccination and COVID‐19 vaccination [17]. Data were deduplicated such that no more than 1 swab per person per 28‐day period was included. Swabs in children 2–17 were further excluded if the vaccination date was unknown. Otherwise, multiple imputation methods were used to account for missing data on vaccination date (adults only), clinical risk status or onset date (numbers of swabs with missing data are given in Figure 1); 20 imputations were carried out. Where it was indicated that an adult patient was vaccinated, but no vaccination date was given, multiple ‘hot deck’ imputation was used to assign vaccination status (within 14 days or 14 days+ ‘fully vaccinated’) of vaccinated patients that were swabbed during the same week. Multiple imputation of clinical risk status was based on a logistic regression model with explanatory variables: interaction between vaccination age eligibility and vaccination at any time during the season, patient age, sex, scheme and influenza result. Onset status (swabbed within 0–7 days of symptom onset or swabbed beyond 7 days) was imputed similarly, with scheme, age, sex and positivity for influenza or any other respiratory virus as explanatory variables. The model for VE then included an interaction between vaccination status (unvaccinated/vaccinated within 14 days/fully vaccinated) and onset status, and VE is reported based on only the OR for influenza positive swab in fully vaccinated versus unvaccinated patients, swabbed within 7 days of onset. Sensitivity analyses were conducted to check departure from estimates based on non‐missing data, differences in VE were no more than 4% and had no impact on interpretation.

To explore longer term effectiveness of influenza vaccines, we estimated the effectiveness (during our 2022/23 study period) of combinations of two season's vaccination: vaccinated 2022/23 only, vaccinated 2021/22 only and vaccinated in both the 2021/22 and 2022/23 seasons, versus unvaccinated both seasons, for all ages. Past season vaccination status was unavailable for Wales, so analyses including 2021/22 were carried out for England and Scotland only. Given that VE may vary by age and type of vaccine received, estimates were population weighted by four broad age bands: 2–17, 18–49, 50–64 and 65+. The population of Great Britain by age group was based on 2021 Office of National Statistics estimates.

VE was estimated for genetically characterized influenza A(H3N2) viruses from England (data unavailable for Scotland and Wales), for all ages with population weighting as above.

To minimise inclusion of underpowered results, VE estimates where any expected cell count of numbers of unvaccinated and vaccinated cases and controls was less than 10 were excluded.

## 3 Results

During the study period, 18,411 swabs were taken in participating sentinel primary care practices, including 9667 through the RCGP RSC, 7737 through PHS and 1074 through PHW. A total of 5331 samples was excluded or did not contribute to main estimates; reasons are summarised in Figure 1. Of the 13,080 swabs contributing to main estimates, 7460 had complete data. The number of cases and controls by age cohort is also provided; in total, there were 10,635 controls and 2445 cases, of whom 1324 were due to A(H3N2), 661 were A(H1N1)dpm09, 219 were influenza A (unknown subtype) and 253 were influenza B, including 12 dual infections.

**FIGURE 2:**
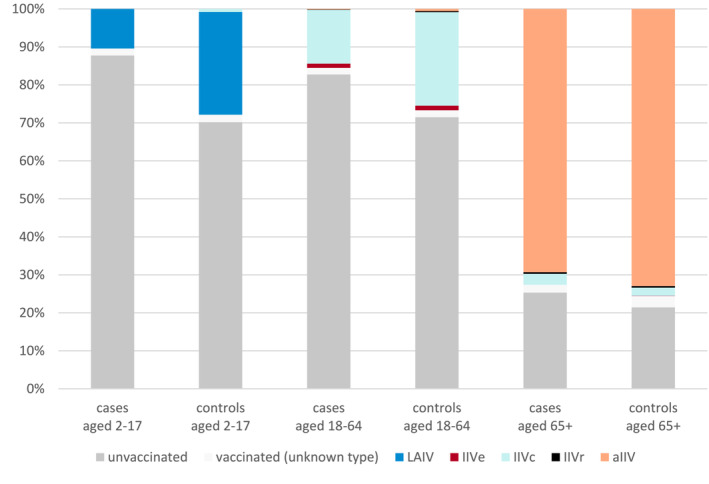
Percentage vaccination status of cases and controls by age cohort. aIIV = adjuvanted quadrivalent vaccine, IIVc = cell culture–based quadrivalent vaccine, IIVe = standard dose egg‐based quadrivalent vaccine, IIVr = recombinant quadrivalent vaccine, LAIV = live attenuated influenza vaccine.

**FIGURE 3:**
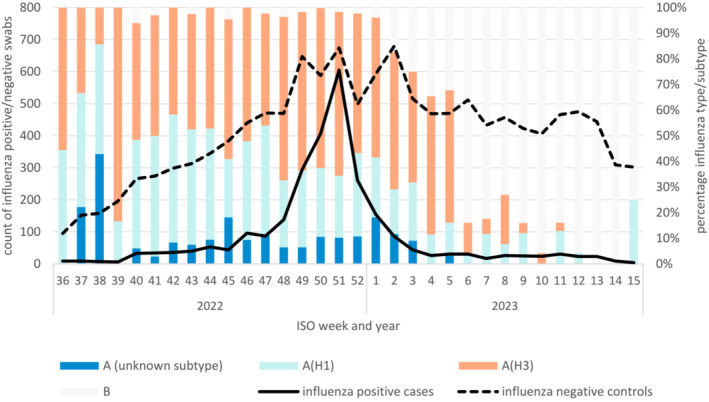
Count of influenza positive cases and influenza negative controls by ISO week swab taken (lines) and the percentage of influenza types or subtypes among influenza positive swabs by ISO week (bars).

Tables of descriptive statistics are given in Data S1. A percentage breakdown of vaccination status for cases and controls at the time of swabbing is given in Figure 2; the predominance of LAIV among vaccinated children, IIVc among 18–64 year olds and aIIV among 65+ year olds is apparent. A plot of the number of cases and controls included in the study by week, including a breakdown of influenza type/subtype, is given in Figure 3. This shows an increase in influenza A(H3N2) and A(H1N1) cases to week 51 in 2022, followed by a sharp drop in case numbers, and then a transition to predominance of influenza B from week 6 in 2023.

### 3.1 Overall VE

The VE estimates stratified by age cohort (2–17, 18–64 and 65+) against all influenza, overall and by vaccine type, influenza A(H1N1)pdm09, A(H3N2) and B, are shown in Table 1. For all influenza, overall VE was 66% (95% CI: 53%–76%) in children aged 2–17, 47% (95% CI: 37%–56%) in adults 18–64 and 30% (95% CI: −6% to 54%) in adults aged 65+. Estimates of overall VE are reflected in VE by the predominant vaccine type for each age cohort. It was only possible to estimate VE for several vaccine types among 18–64 year olds: VE for IIVe was 26% (95% CI: −32% to 58%), for aIIV 76% (95% CI: −8% to 95%) and for IIVc 48% (95% CI: 37%–57%); there was wide uncertainty around the estimates for IIVe and aIIV, and all confidence intervals overlap.

**TABLE 1:** Influenza vaccine effectiveness stratified by age cohort.

	Controls (influenza test negative)^a^		Cases (influenza test positive)^a^		VE^b^ (95% CI)
	Unvacc	Vacc	Unvacc	Vacc	
Ages 2–17					
All vaccinated	1181	502	437	61	66% (53%–76%)
By vaccine type					
LAIV	1181	455	437	52	68% (55%–78%)
By influenza type					
A(H1N1)pdm09	1181	502	85	9	73% (43%–87%)
A(H3N2)	1181	502	288	47	59% (40%–72%)
B	1181	502	44	< 3	95% (62%–99%)
Ages 18–64					
All vaccinated	5001	1994	1412	294	47% (37%–56%)
By vaccine type					
IIVe	5001	83	1412	19	26% (−32% to 58%)
IIVc	5001	1724	1412	242	48% (37%–57%)
aIIV	5001	40	1412	3	76% (−8% to 95%)
By influenza type					
A(H1N1)pdm09	5001	1994	396	91	42% (23%–56%)
A(H3N2)	5001	1994	694	166	37% (21%–50%)
B	5001	1994	190	15	71% (49%–84%)
Ages 65+					
All vaccinated	420	1537	61	180	30% (−6% to 54%)
By vaccine type					
aIIV	420	1428	61	167	29% (−10% to 54%)
By influenza type					
A(H1N1)pdm09	420	1537	21	59	5% (−87% to 52%)
A(H3N2)	420	1537	32	97	35% (−11% to 62%)
B	420	1537	< 3	< 3	Insufficient cases

Abbreviations: CI = confidence interval, unvacc = unvaccinated, vacc = vaccinated.

^a^ Cell counts include those with missing data for onset and vaccination date.

^b^ Adjustments were made for week of sample (spline), age group, scheme and clinical risk status.

By influenza A subtype, VE was 73% (95% CI: 43%–87%), 42% (95% CI: 23%–56%) and 5% (95% CI: −87% to 52%) against influenza A(H1N1)pdm09 among 2–17, 18–64 and 65+ year olds, respectively, and 59% (95% CI: 40%–72%), 37% (95% CI: 21%–50%) and 35% (95% CI: −11% to 62%) against influenza A(H3N2) in the respective age cohorts. For influenza B, VE was higher among 2–17 year olds (VE 95%, 95% CI: 62%–99%) and 18–64 year olds (VE 71%, 95% CI: 49%–84%), while there were very few influenza B detections among those age 65+.

### 3.2 VE by Vaccination Status Over Influenza Seasons 2021/22 and 2022/23

With unvaccinated over two seasons as the reference category, positive VE during the 2022/23 influenza season of the 2021/22 vaccine alone was demonstrated against influenza A(H1N1)pdm09 (VE 44%, 95% CI: 25%–58%), A(H3N2) (VE 25%, 95% CI: 6%–41%) and influenza B (VE 65%, 95% CI: 36%–81%), shown in Table 2. VE was comparable for those vaccinated in 2022/23 only (VE 56%, 95% CI: 42%–67%) and those vaccinated both seasons (VE 57%, 95% CI: 50%–63%). Among individuals who had received the 2021/22 vaccine, incremental effectiveness of the 2022/23 vaccine was 32% (95% CI: 17%–44%) against all influenza; however, results varied by type/subtype: 17% against influenza A(H1N1)pdm09 (95% CI: −16% to 41%), 38% against A(H3N2) (95% CI: 19%–53%) and 52% against influenza B (95% CI: −11% to 79%).

**TABLE 2:** Effectiveness during the 2022/23, of two seasons of influenza vaccination 2021/22 and 2022/23.

	Controls^a^		Cases^a^		VE^b^ with population age weighting (95% CI) (reference: unvaccinated both seasons)	VE^b^ with population age weighting (95% CI) (reference: vaccinated 2021/22 only)
	Unvacc	Vacc	Unvacc	Vacc		
All cases						
2021/22 only	4793	1561	1493	294	37% (26%–47%)	Reference
2022/23 only	4793	581	1493	76	56% (42%–67%)	
2022/23 and 2021/22	4793	3297	1493	428	57% (50%–63%)	32% (17%–44%)
A(H1N1)pdm09						
2021/22 only	4793	1561	394	73	44% (25%–58%)	Reference
2022/23 only	4793	581	394	19	57% (28%–75%)	
2022/23 and 21/22	4793	3297	394	132	54% (40%–65%)	17% (−16% to 41%)
A(H3N2)						
2021/22 only	4793	1561	754	181	25% (6%–41%)	Reference
2022/23 only	4793	581	754	47	46% (19%–65%)	
2022/23 and 2021/22	4793	3297	754	242	54% (42%–63%)	38% (19%–53%)
B						
2021/22 only	4793	1561	210	14	65% (36%–81%)	Reference
2022/23 only	4793	581	210	3	86% (52%–96%)	
2022/23 and 2021/22	4793	3297	210	13	83% (68%–91%)	52% (−11% to 79%)

Abbreviations: CI = confidence interval, unvacc = unvaccinated, vacc = vaccinated.

^a^ Cell counts include those with missing data for onset.

^b^ Adjustments were made for week of sample (spline), age group, scheme and clinical risk status.

### 3.3 Genetic Characterisation

Genetic characterisation of 146 influenza A(H1N1)pdm09 viruses detected in patients presenting to sentinel GPs showed that all belonged within subgroup 6B.1A.5a; 105 (72%) belonged to the emerging clade 5a.2a.1, while 41 (28%) belonged to clade 5a.2a. The Northern Hemisphere (N. Hemisphere) 2022/23 influenza A(H1N1)pdm09 vaccine strains (egg‐based A/Victoria/2570/2019 and cell culture–based A/Wisconsin/588/2019) belong within clade 5a.2. Circulating strains during this period showed genetic heterogeneity, and genetic characterisation of 419 A(H3N2) influenza viruses showed that they all belong to clade 3C.2a1b.2a.2, with 191 (46%) belonging to subclade designated as 2b, 100 (24%) belonging to subclade 2a.1b, 56 (13%) belonging to subclade 2a.3a.1 and between 1 and 10 viruses belonging within each of subclades 2a, 2a.1, 2a.1a, 2a.3 and 2a.3b. The N. Hemisphere 2022/23 influenza A(H3N2) vaccine strains (cell culture–propagated A/Stockholm/5/2021 and egg‐propagated A/Darwin/9/2021) belong in genetic subclade 2a (of clade 3C.2a1b.2a.2). Of 79 influenza B viruses characterised during the season, all fell within the V1A.3a.2 subgroup, in common with the N. Hemisphere 2022/23 B/Victoria‐lineage virus (a B/Austria/1359417/2021‐like virus). No characterised viruses were of B/Yamagata lineage.

VE for A(H3N2) overall and by A(H3N2) subclades is shown in Table 3. VE was 69% (95% CI 45 to 83%) for subclade 2a.1b, 69% (95% CI 24 to 87%) for subclade 2a.3a.1 and 50% (95% CI 23 to 67%) for subclade 2b.

**TABLE 3:** Vaccine effectiveness against A(H3N2).

	Controls^a^		Cases^a^		VE^b^ with population age weighting (95% CI)
	Unvacc	Vacc	Unvacc	Vacc	
A(H3N2), all cases	2377	1395	551	154	50% (37%–61%)
A(H3N2), cases with genetic characterisation	2377	1395	288	72	54% (37%–66%)
Subclade 2a.1b	2377	1395	77	16	69% (45%–83%)
Subclade 2a.3a.1	2377	1395	42	5	69% (24%–87%)
Subclade 2b	2377	1395	136	37	50% (23%–67%)

Abbreviations: CI = confidence interval, unvacc = unvaccinated, vacc = vaccinated.

^a^ Cell counts include those with missing data for onset (all in this analysis have known vaccination date).

^b^ Adjustments were made for week of sample (spline), age group, scheme and clinical risk status.

## 4 Discussion

This paper reports moderate influenza VE during an extraordinary influenza season dominated by A(H3N2) and latterly influenza B, the latter at low levels. We demonstrate moderate effectiveness against influenza A(H3N2) overall and in children aged 2–17 and in adults aged 18–64. In children 2–17 years of age and working age adults aged 18–64, we also found moderate effectiveness against all influenza and influenza A(H1N1)pdm09 and moderate to high effectiveness against influenza B, dependent on age. Our VE estimates against all influenza, influenza A(H1N1)pdm09 and A(H3N2) for adults aged 65+ were positive, but nonsignificant; very little influenza B circulated among this age group.

Findings of higher VE among children mirror 2022/23 interim estimates of VE in primary care and hospital settings from across Europe and the United States [20, 21, 22], but not Canada [23]. Similarly, where interim VE among adults aged 18–64 and 65+ was reported, VE was generally lower among those aged 65+ [20, 22]. Findings of lower VE among the elderly is consistent with findings from earlier seasons, with immunosenescence considered to be a contributing factor [24].

There are several strengths to this study. The test negative case–control design was used, which is a well‐established approach to measure influenza VE. Estimates should be comparable with those reported for the United Kingdom up to 2019/20, while no estimates based on primary care data are available for 2020/21 and 2021/22 seasons due to lack of circulating influenza and interruption of GP sentinel surveillance during the COVID‐19 pandemic. Further, the increased sample size has enabled reasonably precise VE estimates by broad subgroups including by genetic lineages, not previously possible. There are some limitations—numbers were low and 95% CIs wide to permit comparison by vaccine type or by genetic group. Data items were sometimes missing and multiple imputation methods were used; however, sensitivity analyses suggested that this should not have substantially impacted our findings. There were some differences between the three national schemes, including the timing of swabs post onset (7 or 10 days), methods for collection of vaccination histories and subtle differences in acute respiratory infection (ARI) definitions used; these were accounted for by targeting our analysis at swabs taken within 7 days and adjustment for scheme in VE analyses. Vaccination may not be recorded if given privately (e.g., in workplaces), and this is likely to have the most impact on those aged 18 to 49 who were not universally eligible for free vaccination.

We found evidence of overall low to moderate VE this season, particularly against influenza A(H3N2) and A(H1N1)pdm09, while VE estimated against influenza B was moderate to high. Later delivery of the LAIV programme in England may have impacted on the overall efficiency of the vaccination campaign, as A(H3N2) circulated early.

Genetic characterisation of A(H1N1)pdm09 viruses this season showed that in the United Kingdom, they primarily belonged to genetic clade 6B.1A.5a.2a.1. The United Kingdom differed from many European countries, where circulating viruses were dominated by clade 6B.1A.5a.2a; however, our A(H1N1)pdm09 VE findings are broadly similar to the interim VE estimates published earlier in the season from elsewhere in Europe [20]. Published interim VE estimates from the 2022/23 season are lower than those reported for 2021/22 end‐of‐season; for example, 2021/22 end‐of‐season VE and 2022/23 mid‐season VE against A(H1N1)pdm09 were 75% and 28%, respectively, in European multicentre primary care studies and 76% and 26%, respectively, in English emergency care [1, 20, 25]. The antigenic distance between the 6B.1A.5a.2 vaccine viruses (that remained the same in both the northern hemisphere 2021/22 and 2022/23 vaccines) and circulating strains has grown wider since the 2021/22 season. The World Health Organization (WHO) updated the A(H1N1)pdm09 composition for the 2023/24 Northern Hemisphere vaccines to A/Victoria/4897/2022 for egg‐grown vaccines and cell culture–propagated A/Wisconsin/67/2022‐like viruses [26]. Ongoing evaluation of the performance of these new vaccine compositions will be important in the 2023/24 season.

Low VE against influenza A(H3N2) has been seen globally in recent seasons, and a number of explanations have been put forward including antigenic changes in circulating A(H3N2) viruses and egg adaption of the vaccine virus [12]. In 2022/23, genetic characterisation showed A(H3N2) viruses belonging to a number of cocirculating 3C.2a1b.2a.2 subclades (primarily 2b, 2a.3a.1 and 2a.1b); hence, the 2a vaccine virus was challenged to provide protection against an array of circulating strains. Subclade‐specific VE point estimates were higher for subclades 2a.3a.1 and 2a.1b than for 2b, but sample sizes were not large and CIs wide, so these estimates should be interpreted along with those from other countries; interim estimates from Canada saw little difference between viruses with the H156S substitution (e.g., 2a.3a.1 and 2a.1b) and those without (e.g., 2b) [23]. Cell‐based influenza vaccines, which avoid the issue of egg‐adaption, were recommended for adults aged 18–64 in the United Kingdom in 2022/23. IIVc VE was moderate, with a VE point estimate sitting both between that seen for LAIV in children and aIIV in adults aged 65+, and above that seen for IIVe in adults aged 18–64. This finding is consistent with the UK Joint Committee on Vaccination and Immunisation advice on preference for IIVc over egg‐based vaccines (IIVe) for people 18–64 with medical risk conditions.

The vaccine influenza B/Victoria virus was well matched to the circulating strains [26]. Our moderate to high VE estimates against influenza B are in line with mid‐season estimates for other European countries [20]. There was little influenza B circulation among those aged 65+. No B/Yamagata viruses were identified, a finding replicated globally [27].

We found positive effectiveness of 2021/22 season vaccination alone, suggesting that influenza vaccines provide limited long‐term protection. VE estimates were consistently higher if the current season (2022/23) vaccine was received, which concurs with findings from other studies [28] and confirms the value of annual vaccination. The northern hemisphere A(H1N1)pdm09 vaccine strain was the same in the 2022/23 vaccine as the 2021/22 vaccine; hence, waning antibody levels were responsible for the reduced VE compared to the current season. The A(H3N2) vaccine strain was updated from an A/Cambodia/e0826360/2020‐like virus (group 1a of clade 3C.2a1b.2a.1) in 2021/22 to an A/Darwin/9/2021‐like virus in 2022/23 (group 2a of clade 3C.2a1b.2a.2); while the 2021/22 vaccine may have offered some protection, the 2022/23 vaccine is a closer antigenic match to the main circulating strains [26]. The influenza B Victoria vaccine strain was updated from a B/Washington/02/2019‐like virus (group V1A.3) in 2021/22 to a B/Austria/1359417/2021‐like virus (group V1A.3a.2) in 2022/23. The B/Victoria 2021/22 vaccine virus was a poor match to the 2022/23 circulating B Victoria lineage viruses [26]; hence, our finding of moderately high VE against influenza B from the 2021/22 vaccine alone is unexpected.

In summary, this paper provides evidence of moderate influenza VE during an A(H3N2) dominated extraordinary 2022/23 season in the mainland United Kingdom. Furthermore, the youngest age group primarily vaccinated with LAIV encouragingly shows moderate VE with all types and subtypes tested. Lower effectiveness among the population aged 65 and over remains an area of concern that may be addressed by recent and emerging vaccine technologies to overcome immunosenescence. Low to moderate effectiveness against A(H1N1)pdm09 is also a concern, and it will be important to monitor the effectiveness of the updated A(H1N1)pdm09 northern hemisphere vaccine strain in the 2023/24 season.

## Author Contributions

**Heather J. Whitaker:** Conceptualization; Formal analysis; Methodology; Writing – original draft; Writing – review and editing. **Naoma Willam:** Data curation; Writing – review and editing. **Simon Cottrell:** Conceptualization; Data curation; Supervision; Writing – review and editing; Project administration. **Rosalind Goudie:** Data curation; Writing – review and editing. **Nick Andrews:** Conceptualization; Methodology; Supervision; Writing – review and editing. **Josie Evans:** Conceptualization; Data curation; Supervision; Writing – review and editing. **Catherine Moore:** Investigation; Supervision; Writing – review and editing; Project administration. **Utkarsh Agrawal:** Conceptualization; Writing – review and editing. **Katie Hassell:** Conceptualization; Data curation; Writing – review and editing. **Rory Gunson:** Investigation; Supervision; Writing – review and editing. **Jana Zitha:** Data curation; Writing – review and editing. **Sneha Anand:** Project administration; Writing – review and editing. **Praveen Sebastian‐Pillai:** Data curation; Writing – review and editing. **Panoraia Kalapotharakou:** Data curation; Writing – review and editing. **Cecilia Okusi:** Data curation; Writing – review and editing. **Katja Hoschler:** Investigation; Supervision; Writing – review and editing. **Gavin Jamie:** Data curation; Writing – review and editing. **Beatrix Kele:** Data curation; Investigation; Writing – review and editing. **Mark Hamilton:** Data curation; Writing – review and editing. **Anastasia Couzens:** Investigation; Writing – review and editing. **Catherine Quinot:** Data curation; Writing – review and editing. **Kathleen Pheasant:** Investigation; Writing – review and editing. **Rachel Byford:** Data curation; Writing – review and editing. **Kimberly Marsh:** Project administration; Writing – review and editing; Supervision. **Chris Robertson:** Conceptualization; Methodology; Supervision; Writing – review and editing. **Simon de Lusignan:** Conceptualization; Project administration; Supervision; Writing – review and editing. **Christopher Williams:** Conceptualization; Writing – review and editing; Project administration; Supervision. **Maria Zambon:** Supervision; Conceptualization; Writing – review and editing; Investigation; Project administration. **Jim McMenamin:** Conceptualization; Project administration; Supervision; Writing – review and editing. **Conall H. Watson:** Conceptualization; Project administration; Supervision; Writing – original draft; Writing – review and editing.

## Ethics Statement

UK public health agencies have permission to process patient confidential information for national surveillance of communicable diseases under: Regulation 3 of the Health Service Regulation 2002 for England, the Public Health (Scotland) Act 2008 and the NHS Scotland Act 1978 for Scotland, and the Public Health Wales National Health Service Trust (Establishment) Order 2009 for Wales. The work was reviewed by the UKHSA Research Ethics and Governance Group who confirmed the work was covered by Regulation 3 of The Health Service (Control of Patient Information) Regulations 2002, therefore specific ethical approval was not necessary. This review was also undertaken in each UK country.

## Conflicts of Interest

SdeL, within his academic role, is the director of the RCGP RSC. He has received grants through his University from AstraZeneca, GSK, Moderna, Sanofi and Seqirus for vaccine‐related research and been a member of advisory boards for AstraZeneca, GSK, Sanofi and Seqirus. HW and CHW's department has received cost‐recovery payment from CSL Seqirus for analysis undertaken for regulatory review.

### Peer Review

The peer review history for this article is available at https://www.webofscience.com/api/gateway/wos/peer‐review/10.1111/irv.13295.

## Supporting information


**Table S1** Descriptive statistics.

## Data Availability

Data supporting this study cannot be made available due to ethical and legal reasons.
