# Supplementary material for: End of 2022/23 Season Influenza Vaccine Effectiveness in Primary Care in Great Britain
Source: Influenza Other Respir Viruses. 2024 May 14;18(5):e13295. doi: 10.1111/irv.13295 (PMC11093773; doi:10.1111/irv.13295)
Supplement: Supplementary file 1 — Table S1 Descriptive statistics. [file IRV-18-e13295-s001.docx]

### Supplementary material

### S1 Descriptive statistics

Table S1.1 Counts of influenza cases and controls by demographic and health characteristics, month sampled, and influenza vaccination status in children aged 2 to 17

|  |  | A(unknown subtype) | A(H1N1)^1^ | A(H3N2)^1^ | B | control | positivity |
| --- | --- | --- | --- | --- | --- | --- | --- |
| total |  | 27 | 94 | 335 | 45 | 1683 | 23% |
| age | 2 to 3 | 5 | 17 | 38 | 4 | 391 | 14% |
|  | 4 to 6 | 6 | 20 | 63 | 5 | 459 | 17% |
|  | 7 to 10 | 3 | 19 | 94 | 8 | 360 | 25% |
|  | 11 to 13 | 11 | 18 | 73 | 13 | 230 | 33% |
|  | 14 to 17 | <3 | 20 | 67 | 15 | 243 | 30% |
| sex | F | 13 | 48 | 154 | 15 | 848 | 21% |
|  | M | 14 | 46 | 181 | 30 | 835 | 24% |
| clinical risk status | none | 26 | 84 | 290 | 36 | 1,439 | 23% |
|  | at risk | <3 | 10 | 39 | 8 | 226 | 20% |
|  | unknown | 0 | 0 | 6 | <3 | 18 | 28% |
| scheme | England | 27 | 88 | 316 | 44 | 1,591 | 23% |
|  | Wales | 0 | 6 | 19 | <3 | 92 | 21% |
| month sampled | Sep | <3 | 0 | <3 | 0 | 78 | 3% |
|  | Oct | 0 | 10 | 22 | <3 | 167 | 17% |
|  | Nov | 3 | 23 | 56 | 5 | 279 | 24% |
|  | Dec | 23 | 58 | 243 | 14 | 378 | 47% |
|  | Jan | 0 | <3 | 11 | 7 | 219 | 8% |
|  | Feb | 0 | <3 | <3 | 8 | 230 | 4% |
|  | Mar | 0 | 0 | <3 | 10 | 285 | 4% |
|  | Apr | 0 | 0 | 0 | 0 | 47 | 0% |
| flu vaccination status | unvaccinated | 23 | 85 | 288 | 44 | 1,181 | 27% |
|  | vacc (unknown type) | 0 | 0 | 9 | 0 | 33 | 21% |
|  | LAIV | 4 | 9 | 38 | <3 | 455 | 10% |
|  | IIVc | 0 | 0 | 0 | 0 | 14 | 0% |
| flu vaccination status over 2 seasons | unvacc both | 14 | 59 | 198 | 40 | 752 | 29% |
|  | vacc 21/22 only | 9 | 20 | 74 | 3 | 360 | 22% |
|  | vacc 22/23 only | <3 | 4 | 11 | <3 | 150 | 11% |
|  | vacc 22/23 & 21/22 | <3 | 5 | 33 | 0 | 329 | 11% |

^1^includes 3 A(H1N1)+A(H3N2) co-infections

LAIV = live attenuated influenza vaccine, IIVc = cell-based quadrivalent influenza vaccine, vacc = vaccinated, unvacc = unvaccinated

Table S1.2 Counts of influenza cases and controls by demographic and health characteristics, month sampled, and influenza vaccination status in adults aged 18 to 64

|  |  | A(unknown subtype) | A(H1N1)^1^ | A(H3N2)^1^ | B^1^ | control | positivity |
| --- | --- | --- | --- | --- | --- | --- | --- |
| total |  | 163 | 487 | 860 | 205 | 6995 | 20% |
| age | 18to34 | 59 | 139 | 409 | 135 | 2576 | 22% |
|  | 35to49 | 58 | 218 | 273 | 58 | 2218 | 21% |
|  | 50to64 | 46 | 130 | 178 | 12 | 2201 | 14% |
| sex | F | 104 | 309 | 598 | 125 | 4847 | 19% |
|  | M | 59 | 178 | 262 | 80 | 2148 | 21% |
| clinical risk status | none | 41 | 210 | 396 | 119 | 2438 | 24% |
|  | at risk | 22 | 93 | 183 | 18 | 1573 | 17% |
|  | unknown | 100 | 184 | 281 | 68 | 2984 | 17% |
| scheme | England | 31 | 152 | 342 | 94 | 2635 | 19% |
|  | Scotland | 132 | 300 | 445 | 99 | 4113 | 19% |
|  | Wales | 0 | 35 | 73 | 12 | 247 | 33% |
| month sampled | Sep | 3 | 7 | 10 | 0 | 353 | 5% |
|  | Oct | 7 | 42 | 34 | 3 | 645 | 12% |
|  | Nov | 20 | 76 | 81 | <3 | 931 | 16% |
|  | Dec | 105 | 297 | 609 | 9 | 1304 | 44% |
|  | Jan | 28 | 51 | 108 | 32 | 1277 | 15% |
|  | Feb | 0 | 6 | 17 | 60 | 1008 | 8% |
|  | Mar | 0 | 7 | <3 | 88 | 1110 | 8% |
|  | Apr | 0 | <3 | 0 | 11 | 367 | 3% |
| flu vaccination status | unvaccinated | 141 | 396 | 694 | 190 | 5001 | 22% |
|  | vacc (unknown type) | 0 | 8 | 18 | 3 | 128 | 18% |
|  | IIVe | 0 | 6 | 13 | 0 | 83 | 19% |
|  | IIVc | 21 | 75 | 134 | 12 | 1724 | 12% |
|  | IIVr | 0 | 0 | <3 | 0 | 19 | 5% |
|  | aIIV | <3 | <3 | 0 | 0 | 40 | 7% |
| flu vaccination status over 2 seasons | unvacc both | 124 | 326 | 539 | 170 | 3863 | 23% |
|  | 21/22 only | 17 | 42 | 97 | 10 | 973 | 15% |
|  | 22/23 only | 5 | 13 | 32 | <3 | 380 | 12% |
|  | 22/23 & 21/22 | 17 | 71 | 119 | 11 | 1532 | 12% |

^1^includes 7 A(H1N1)+A(H3N2) and 2 A(H3N2)+B co-infections

IIVe = egg-based standard dose quadrivalent influenza vaccine, IIVc = cell-based quadrivalent influenza vaccine, IIVr = recombinant quadrivalent influenza vaccine, aIIV = adjuvanted quadrivalent influenza vaccine, vacc = vaccinated, unvacc = unvaccinated

Table S1.3 Counts of influenza cases and controls by demographic and health characteristics, month sampled, and influenza vaccination status in adults aged 65 and over

|  |  | A(unknown subtype) | A(H1N1) | A(H3N2) | B | control | positivity |
| --- | --- | --- | --- | --- | --- | --- | --- |
| total |  | 29 | 80 | 129 | 3 | 1957 | 11% |
| age | 65to74 | 14 | 46 | 85 | <3 | 1105 | 12% |
|  | 75+ | 15 | 34 | 44 | <3 | 852 | 10% |
| sex | F | 14 | 45 | 79 | <3 | 1241 | 10% |
|  | M | 15 | 35 | 50 | <3 | 716 | 12% |
| clinical risk status | none | <3 | 7 | 26 | <3 | 267 | 12% |
|  | at risk | 9 | 31 | 47 | 0 | 777 | 10% |
|  | unknown | 18 | 42 | 56 | <3 | 913 | 11% |
| scheme | England | 4 | 25 | 53 | <3 | 743 | 10% |
|  | Scotland | 25 | 53 | 68 | <3 | 1150 | 11% |
|  | Wales | 0 | <3 | 8 | 0 | 64 | 14% |
| month sampled | Sep | 0 | 5 | <3 | 0 | 75 | 7% |
|  | Oct | <3 | 8 | 6 | 0 | 156 | 9% |
|  | Nov | 6 | 7 | 14 | 0 | 275 | 9% |
|  | Dec | 12 | 47 | 77 | <3 | 377 | 27% |
|  | Jan | 9 | 13 | 25 | <3 | 366 | 12% |
|  | Feb | 0 | 0 | 5 | 0 | 297 | 2% |
|  | Mar | 0 | 0 | <3 | <3 | 298 | 1% |
|  | Apr | 0 | 0 | 0 | 0 | 113 | 0% |
| flu vaccination status | unvaccinated | 7 | 21 | 32 | <3 | 420 | 13% |
|  | vacc (unknown type) | 0 | <3 | 4 | 0 | 58 | 8% |
|  | IIVe | 0 | 0 | 0 | 0 | <3 | 0% |
|  | IIVc | 0 | <3 | 5 | 0 | 42 | 14% |
|  | IIVr | 0 | 0 | <3 | 0 | 8 | 11% |
|  | aIIV | 22 | 56 | 87 | <3 | 1428 | 10% |
| flu vaccination status over 2 seasons | unvacc both | 5 | 9 | 17 | 0 | 178 | 15% |
|  | 21/22 only | <3 | 11 | 10 | <3 | 228 | 10% |
|  | 22/23 only | 0 | <3 | 4 | 0 | 51 | 11% |
|  | 22/23 & 21/22 | 22 | 56 | 90 | <3 | 1436 | 11% |

IIVe = egg-based standard dose quadrivalent influenza vaccine, IIVc = cell-based quadrivalent influenza vaccine, IIVr = recombinant quadrivalent influenza vaccine, aIIV = adjuvanted quadrivalent influenza vaccine, vacc = vaccinated, unvacc = unvaccinated
